# A small regulatory RNA controls antibiotic adaptation in *Staphylococcus aureus* by modulating efflux pump expression

**DOI:** 10.1128/aac.01176-24

**Published:** 2025-04-03

**Authors:** Kam Pou Ha, Etornam Kofi Kumeko, Philippe Bouloc

**Affiliations:** 1 Université Paris-Saclay, CEA, CNRS, Institut de Biologie Intégrative de la Cellule (I2BC) 27048, Gif-sur-Yvette, Île-de-France, France; The Peter Doherty Institute for Infection and Immunity, Melbourne, Victoria, Australia

**Keywords:** *Staphylococcus aureus*, small regulatory RNA, RsaA, fitness, antibiotic susceptibility, norfloxacin, ciprofloxacin, cefazolin, β-bactam antibiotics, MgrA

## Abstract

*Staphylococcus aureus* is an opportunistic pathogen that poses a considerable burden to healthcare settings worldwide, aided by its ability to thrive in different environmental growth conditions and survive exposure to antibiotics. Small regulatory RNAs (sRNAs) are decisive in enhancing bacterial fitness by modulating gene expression in response to changing environmental conditions. We investigated the role of sRNAs in the adaptation of *S. aureus* to antibiotics. By assessing the fitness of a library of sRNA mutants, we identified that RsaA sRNA is required for optimal bacterial growth when exposed to low concentrations of fluoroquinolone, a class of antibiotics targeting DNA replication. We also found that in the absence of RsaA, *S. aureus* is less susceptible to β-lactam antibiotics, which act on the cell wall. RsaA has been reported to prevent the expression of MgrA, a master regulatory protein controlling the expression of efflux pumps. Here, we show that RsaA affects the sensitivity of *S. aureus* to fluoroquinolone and β-lactam antibiotics through MgrA. RsaA has two forms, a short one commonly referred to in RsaA studies, and a long form about twice the length, of which less is known. Interestingly, our phenotype was only restored when complemented with the long form of the gene or when it was supplied in two parts, the short form and the missing part to obtain the long form. This work demonstrates the role of regulatory RNAs in the adaptation of *S. aureus* to antibiotic resistance and highlights their value as potential therapeutic targets for manipulating individual sRNA responses to promote the efficacy of existing antibiotics.

## INTRODUCTION

Antibiotic resistance refers to the ability of bacteria to grow in antibiotic concentrations that are considered inhibitory (1). Resistance involves the acquisition of resistance genes from other bacteria or by genetic mutation (1–3), leading to mechanisms such as enzymatic inactivation of antibiotics, alteration of antibiotic targets, or efflux pumps that expel antibiotics from bacterial cells (2, 3). By contrast, antibiotic tolerance is the prolonged survival of bacteria to bactericidal antibiotics for extended periods without necessarily acquiring genetic changes (1, 4). In an extreme subset of tolerance, also called persistence, bacterial cells may enter a dormant or near-dormant state to protect cellular processes against the action of antibiotics (5). Antibiotic persistence and tolerance, collectively termed recalcitrance, represent transient phenotypic changes in the bacterial population that increase survival in the presence of usually lethal antibiotic concentrations (6). Recalcitrance has been demonstrated as a prime starting point for the development of antibiotic resistance (7–9).

Although antibiotic resistance mechanisms have been well studied, global efforts have primarily focused on methicillin-resistant *Staphylococcus aureus*, which represent only a subset of the overall *S. aureus* burden (10). Indeed, treatment failure is common even when *S. aureus* isolates are sensitive to antibiotics (6), and both susceptible and resistant *S. aureus* infections lead to prolonged illness and increased mortality rates (10, 11). Therefore, understanding how *S. aureus* modulates its sensitivity to antibiotics in the absence of resistance could help in the development of novel treatments to reduce the incidence of both resistance and recalcitrance.

Small regulatory RNAs (sRNAs) are key players in the post-transcriptional regulation of target genes (12). They enable rapid environmental adaptation by base-pairing with their target mRNAs to modulate mRNA translation and stability (12). sRNAs act on almost all cellular functions and as a result, have emerged as important regulators of virulence (13), metabolic adaptation (14) and antibiotic resistance (15, 16). Although many sRNAs have been identified in *S. aureus*, their functions and targets are still mostly unknown (17, 18). The best-described sRNA in *S. aureus* is RNAIII, a large 514-nucleotide sRNA that is involved in virulence (19). Other described sRNAs include RsaE, which is associated with the TCA cycle (20, 21) and arginine catabolism (14), and IsrR, which plays a key role in the iron-sparing response (22, 23). Although sRNAs play important roles in regulating antibiotic-resistance processes such as cell envelope modifications and drug efflux pumps in gram-negative species (15, 24–26), their impact on antibiotic resistance and recalcitrance in gram-positive species has received less attention (15, 16). In addition, the role of sRNAs in antibiotic adaptation is poorly documented.

To find sRNAs required for *S. aureus* to adapt to sub-lethal antibiotic concentrations, we used a fitness assay with sRNA mutants grown in the presence of the fluoroquinolone antibiotic norfloxacin. We identified a single sRNA, RsaA, the loss of which increased sensitivity of the bacterium to fluoroquinolone antibiotics. Further tests revealed that the loss of RsaA also decreased the sensitivity of *S. aureus* to β-lactam antibiotics and that both effects were mediated by RsaA via the master regulatory protein MgrA. Taken together, our results demonstrate that sRNAs contribute toward antibiotic adaptation in *S. aureus*.

## RESULTS

### RsaA is required for growth in the antibiotic norfloxacin

To determine whether sRNAs are required for *S. aureus* to adapt to the presence of the fluoroquinolone antibiotic norfloxacin, we employed a well-established technique that enables the competitive fitness of a sRNA mutant library to be assessed (17, 22, 27, 28). This technique, referred to as the fitness competition assay, uses a library of DNA-tagged deletion mutants in the HG003 strain (Table S1) and enables subtle phenotypes to be screened more easily. Three mutants were independently constructed for each sRNA gene, which was then used to establish three independent libraries (Table S4). Each library was restricted to 48 mutants of “*bona fide*” sRNAs, defined as those that are genetically independent with their own promoter and terminator, to prevent any interference from nearby coding sequences (17). sRNA gene sequences were replaced with specific DNA tags to enable each mutant to be identified and the proportion of each mutant within the total population to be calculated. Using indexed PCR primer pairs, up to 40 samples could be tested in one DNA-seq run (17). Mutants that disappeared or accumulated under a given stress condition indicated a potential role for the corresponding sRNAs under this growth condition.

**Fig 1:**
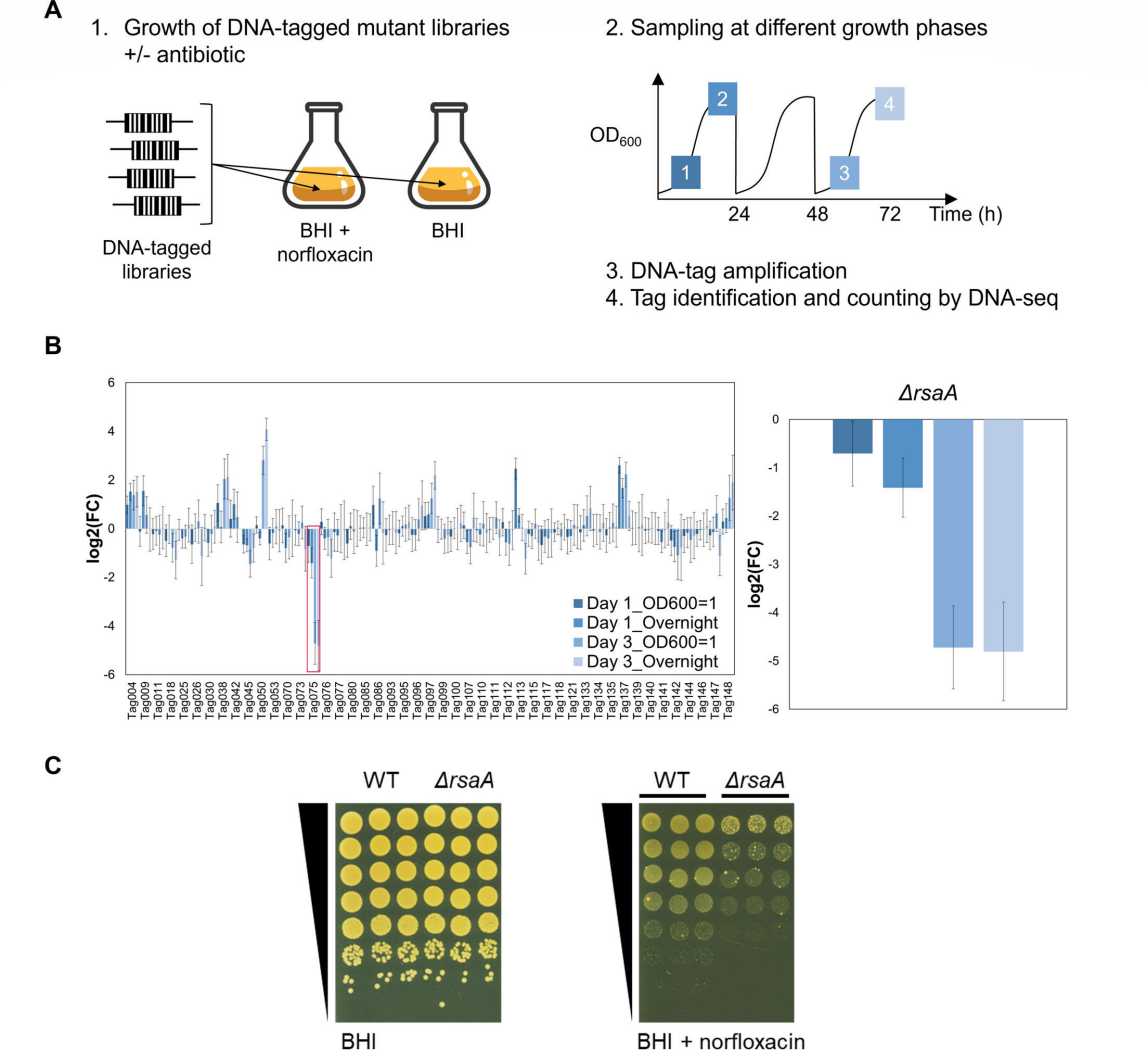
*S. aureus* RsaA is required for optimal growth in the presence of norfloxacin. (A) Experimental protocol scheme to identify mutants with altered fitness in media containing the antibiotic norfloxacin. (B) Evolution of mutant proportions in libraries (for composition, see Table S4) grown in the presence of 0.25 µg/mL norfloxacin (0.5× MIC, see Table S5; Fig. S1 for MIC and growth data) normalized to the same libraries grown in the absence of antibiotic. Error bars indicate the standard deviation from three independent libraries. Left: data with the complete 48 mutant libraries. Tag075 corresponding to the *ΔrsaA* mutant is highlighted by a red box. For Tag-mutant association, see Table S1. Right: selected data for *ΔrsaA* are shown. Color code corresponds to the sampling time color from Fig. 1A. (C) Growth of WT and *ΔrsaA* strains serially diluted 10-fold and spotted onto BHI agar ±0.6 µg/mL norfloxacin (*n* = 3). BHI, Brain Heart Infusion; WT, wild-type.

The sRNA libraries were exposed to a sub-lethal concentration of norfloxacin (0.25 µg/mL at 0.5× MIC, see Table S5; Fig. S1) and grown over 3 days with two serial dilutions. Samples were taken at different time points and growth stages, and the proportion of each mutant within the population was determined (Fig. 1A). Results were normalized to the same inoculum grown in the same medium without antibiotic and sampled at the same growth phases. Among the 48 tested mutants, the strain containing Tag075 displayed the greatest fitness disadvantage, decreasing by 28-fold [log2(FC)= −4.8] in the presence of norfloxacin after 72 h, when compared to the control condition (Fig. 1B). This strain, in which the *rsaA* gene has been replaced by Tag075 (*ΔrsaA*), progressively disappeared from the library under the selective pressure of norfloxacin under sub-lethal concentrations. Neither *rnaIII*, *rsaC*, *rsaD*, *rsaE*, *rsaG*, *isrR* nor *rsaOG* mutants (17), present in the libraries tested, were impacted by the presence of norfloxacin, indicating that their corresponding sRNAs are unlikely to contribute to adaptation to norfloxacin under the tested conditions.

To assess whether the sensitivity of the *rsaA* mutant to norfloxacin also occurred in the absence of the 47 other sRNA strains, monocultures of *ΔrsaA* were grown on agar plates containing norfloxacin. Results from these spot tests revealed that *ΔrsaA* is 100-fold more sensitive to norfloxacin when compared to its parental strain HG003, referred to here as the wild-type (WT) strain (Fig. 1C). In contrast, no difference in growth was observed between either strain when grown in the absence of the antibiotic. Therefore, the loss of RsaA leads to greater sensitivity of *S. aureus* to norfloxacin, and this is independent of any effects on growth in a mixed culture.

Taken together with the fitness competition results, these findings suggested that the RsaA sRNA is required for optimal growth of *S. aureus* in the presence of norfloxacin.

### *S. aureus rsaA* norfloxacin-dependent phenotype is complemented only by the long form of the *rsaA* gene

**Fig 2:**
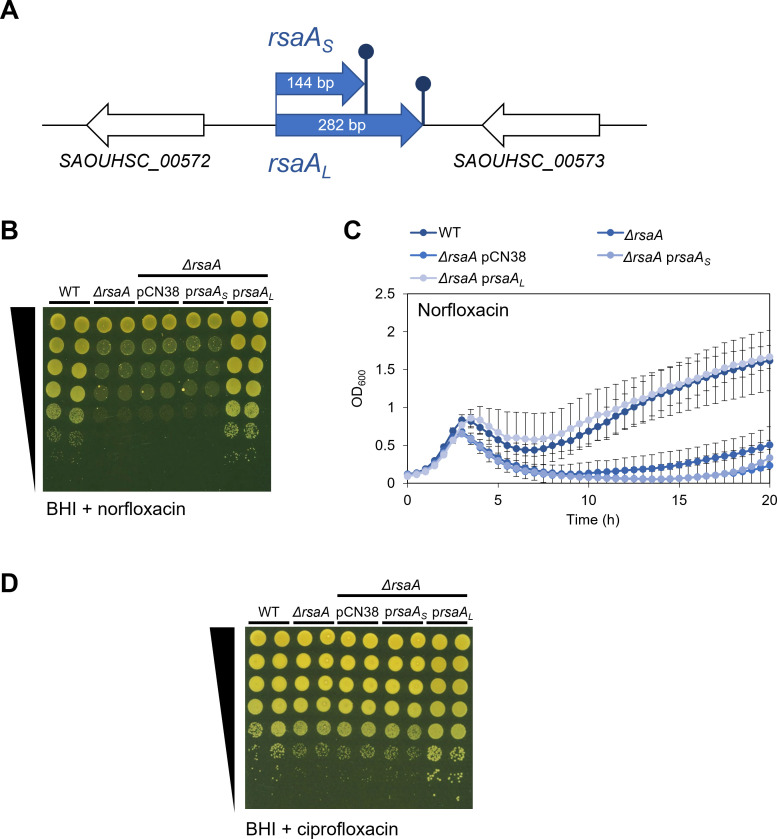
The long form of RsaA restores the wild-type (WT) phenotype, but not the short form. (A) Schematic diagram of long and short forms of RsaA in *S. aureus*. Blue and white arrows represent genes, and dark blue sticks represent transcription terminators. Gene annotations refer to NCTC8325 nomenclature (CP00025.1) retrieved from Genbank (31). The predicted secondary structure of RsaA_L_, along with the experimentally confirmed structure of RsaA_S_ (30), can be found in Fig. S2. (B) Growth of WT, *ΔrsaA*, and *ΔrsaA* containing pCN38 (empty vector), pRsaA_S_ (*rsaA* short form), or pRsaA_L_ (*rsaA* long form), serially diluted 10-fold and spotted onto BHI agar +0.6 µg/mL norfloxacin (*n* = 3). Spot tests in BHI broth alone are shown in Fig. S3. (C) Growth curves of WT, *ΔrsaA,* and *ΔrsaA* derivatives as in (B) grown in BHI broth containing 0.5 µg/mL norfloxacin (*n* = 3). OD_600_ measurements in BHI broth alone are shown in Fig. S3. Error bars represent the standard deviation of the mean. (D) Growth of WT, *ΔrsaA* mutant, empty vector (pCN38), and mutants complemented with RsaA short form (pRsaA_S_) or long form (pRsaA_L_), serially diluted 10-fold and spotted onto BHI agar +0.15 µg/mL ciprofloxacin (*n* = 3). BHI, Brain Heart Infusion.

To confirm that the increased sensitivity of the *rsaA* mutant to norfloxacin was solely due to the loss of RsaA, the *ΔrsaA* mutant was transformed with a plasmid containing a WT copy of the gene. Since there are two versions of the RsaA transcript, a short form of 144 base pairs in length and a long version of 282 base pairs (29, 30) (Fig. 2A; Fig. S2), individual plasmids were constructed to reflect complementation with each form of the gene: pRsaA_L_ and pRsaA_S_ for the long and short forms, respectively.

When the strains were spotted onto agar containing norfloxacin, the loss of RsaA reduced the growth of *S. aureus* by 100-fold when compared to the WT strain, but no improvement in growth occurred when the *ΔrsaA* mutant was transformed with a plasmid containing the short form of the gene (pRsaA_S_), indicating that RsaA_S_ expressed from pRsaA_S_ is not enough to restore the WT phenotype (Fig. 2B). This phenotype was also observed when the same strains were grown in liquid media containing norfloxacin (Fig. 2C), with the difference that growth of *ΔrsaA* was 3.12-fold slower than the WT strain after 20 h rather than 100-fold, which reflects the higher sensitivity of spot tests when compared to growth curves. Unexpectedly, we found that all the cultures followed unusual growth kinetics, in that the cells died after 4 h and recovered after 7 h (Fig. 2C), which did not occur when the strains were grown without norfloxacin (Fig. S3). Given that norfloxacin has been reported to induce prophage activity in other bacterial species (32–34), it is plausible that similar activity could occur in *S. aureus*, particularly since the *S. aureus* HG003 strain contains three prophages: Φ11, Φ12, and Φ13 (35). Nevertheless, in both solid and liquid cultures, complementation of *ΔrsaA* with pRsaA_L_ restored the WT phenotype, demonstrating that the long form of the gene (*rsaA_L_*) is required for optimal growth of *S. aureus* in this antibiotic.

### Loss of RsaA sensitizes *S. aureus* to ciprofloxacin

Norfloxacin belongs to the fluoroquinolone class of antibiotics that cause DNA damage by blocking DNA replication (36), which leads to cell death. To determine whether the phenotype was specific to norfloxacin or could also be observed in another antibiotic of the same functional class, the spot test experiment was repeated with ciprofloxacin. As observed in Fig. 2D, the *ΔrsaA* mutant was more sensitive to ciprofloxacin than the WT strain, and this phenotype was restored when complemented with the long form of the gene. Therefore, RsaA_L_, provided here in multicopy form through pRsaA_L_, leads to a phenotype that affects fluoroquinolones in general.

Combined, these data indicate that RsaA is involved in protecting *S. aureus* against the action of fluoroquinolone antibiotics and that this function requires the long form of the gene to be present.

### Loss of RsaA reduces sensitivity of *S. aureus* to β-lactam antibiotics

Having demonstrated that RsaA is required for the optimal growth of *S. aureus* in the presence of fluoroquinolone antibiotics, we tested whether a similar effect could occur for other antibiotic classes. For this, the β-lactam and glycopeptide antibiotic classes, represented by cefazolin and vancomycin respectively, were selected for testing due to their clinical relevance.

**Fig 3:**
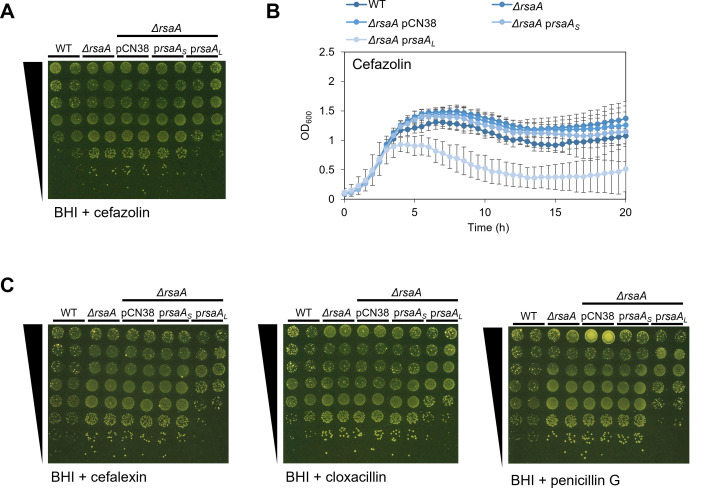
The *ΔrsaA* mutant is less sensitive to β-lactam antibiotics when compared to the WT (A) Growth of WT, *ΔrsaA*, and *ΔrsaA* containing pCN38 (empty vector), pRsaA_S_ (*rsaA* short form), or pRsaA_L_ (*rsaA* long form), serially diluted 10-fold and spotted onto BHI agar +0.3 µg/mL cefazolin (*n* = 3). Spot tests in BHI broth alone are shown in Fig. S3. (B) Growth curves of WT, *ΔrsaA,* and *ΔrsaA* derivatives as in (A) grown in BHI broth containing 0.125 µg/mL cefazolin (*n* = 3). OD_600_ measurements in BHI broth alone are shown in Fig. S3. Error bars represent the standard deviation of the mean. (C) Growth of WT, *ΔrsaA* mutant, and *ΔrsaA* derivatives as in (A), serially diluted 10-fold and spotted onto BHI agar +0.6 µg/mL cefalexin, 0.15 µg/mL cloxacillin, or 0.03125 µg/mL penicillin G (*n* = 3). Spot tests in BHI broth alone are shown in Fig. S3. BHI, Brain Heart Infusion; WT, wild-type.

When the strains were grown in the presence of these two antibiotics, it was revealed that vancomycin had no effect on the growth of the *ΔrsaA* mutant when compared to the WT strain (Fig. S4). By contrast, in the presence of cefazolin, *S. aureus* growth was enhanced in the absence of RsaA by 10-fold in spot tests (Fig. 3A) and by 1.2-fold in liquid culture after 20 h (Fig. 3B), when compared to the WT strain. Furthermore, the WT phenotype was restored only when *ΔrsaA* was complemented by the long form of the gene.

As a follow-up, we repeated the spot tests on a selection of other β-lactam antibiotics: cefalexin, cloxacillin, and penicillin G. Results demonstrated that the growth of the *ΔrsaA* mutant is increased in the presence of β-lactams when compared to the WT strain (Fig. 3C). Taken together, these data demonstrate that the loss of RsaA reduces the sensitivity of *S. aureus* to β-lactam antibiotics. This is opposite to the phenotype found for fluoroquinolone antibiotics, in which the loss of RsaA made *S. aureus* more sensitive to these antibiotics instead. Nevertheless, in both cases, the long form of RsaA is required for this process.

### RsaA acts on MgrA to fully mediate its effect on norfloxacin and partly for cefazolin

RsaA is known to repress the translation of the master regulatory protein, MgrA (30). The latter regulates *S. aureus* virulence by promoting genes involved in capsule synthesis and inhibiting genes related to biofilm formation (37, 38). Separately, MgrA has also been shown to act on several efflux pumps, including the fluoroquinolone efflux pumps NorA (39, 40), NorB (40–42), and NorC (43), and a β-lactam efflux pump, AbcA (42). Since MgrA is reported to inhibit these Nor efflux pumps and promote AbcA (39–43), this would align with our observed phenotypes in the *ΔrsaA* mutant, in which MgrA is no longer being repressed. To test this hypothesis, we performed spot tests on single and double mutants of *ΔrsaA* and *ΔmgrA* in the presence of norfloxacin or cefazolin.

**Fig 4:**
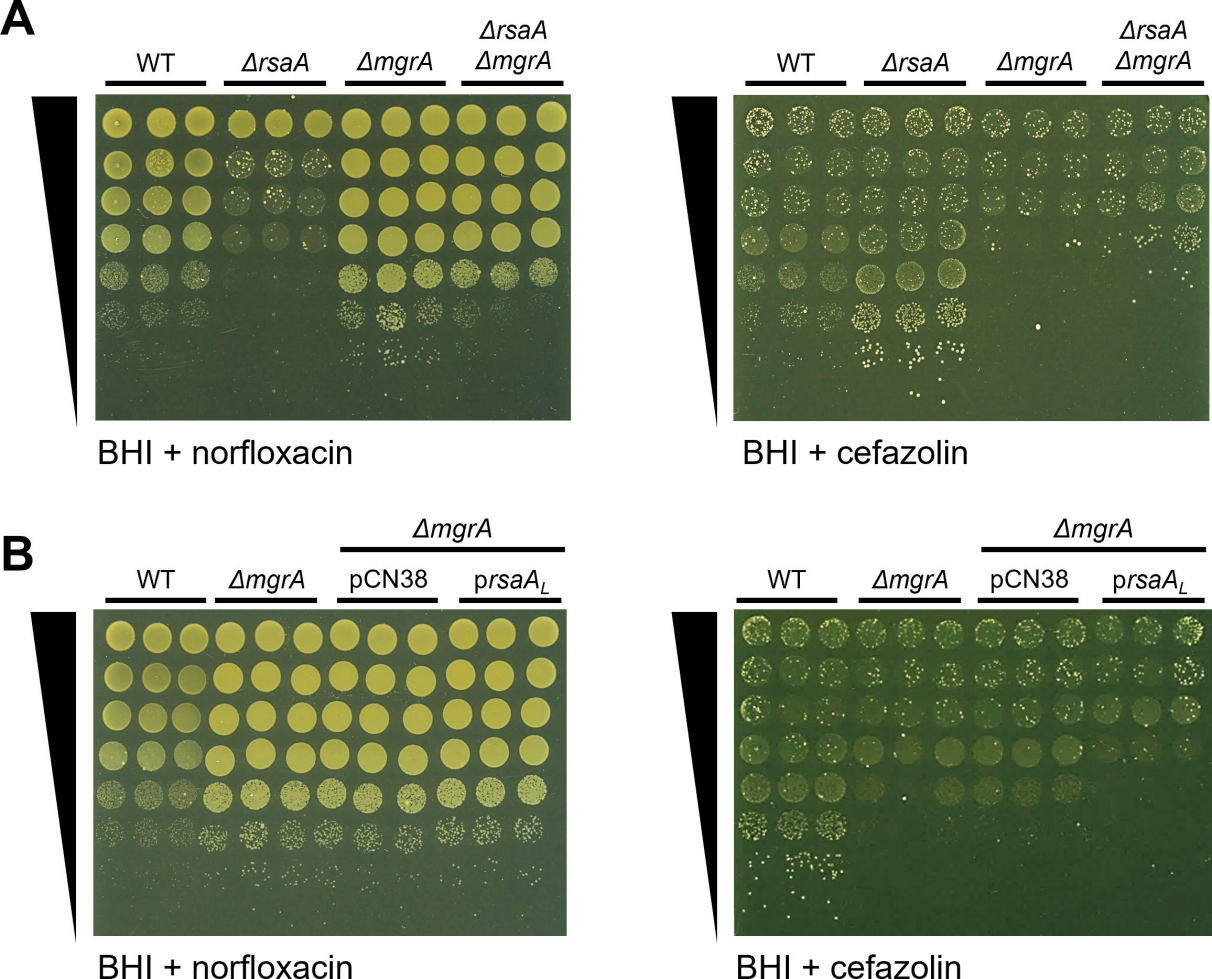
The increased sensitivity of the *ΔrsaA* mutant to norfloxacin is mediated by MgrA. Growth of WT, *ΔrsaA*, *ΔmgrA,* and *ΔrsaA ΔmgrA* strains, serially diluted 10-fold and spotted onto BHI agar containing (A) 0.6 µg/mL norfloxacin or (B) 0.3 µg/mL cefazolin (*n* = 3). (B) Growth of WT, *ΔmgrA,* and *ΔmgrA* complemented with empty vector (pCN38) or long form (pRsaA_L_), serially diluted 10-fold and spotted onto BHI agar containing (C) 0.6 µg/mL norfloxacin or (D) 0.3 µg/mL cefazolin (*n* = 3). Spot tests in BHI alone are shown in Fig. S5. BHI, Brain Heart Infusion; WT, wild-type.

As shown in Fig. 4A, the loss of RsaA and MgrA led to opposite effects on *S. aureus* growth when compared to the WT strain, supporting previous reports of RsaA as a repressor of MgrA. While *ΔrsaA* was more sensitive to norfloxacin than WT *S. aureus*, the *ΔmgrA* mutant showed reduced sensitivity, and the reverse was true of these two strains concerning cefazolin. When the double mutant was tested, its growth in norfloxacin was restored to WT levels, indicating that the increased sensitivity of *ΔrsaA* was most likely caused by derepression of MgrA. In the presence of cefazolin, the growth of *ΔrsaA ΔmgrA* was very slightly increased compared to *ΔmgrA* but did not reach *ΔrsaA* levels, again indicating that the phenotype is likely due to the derepression of MgrA. However, in this case, growth also did not reach WT levels, suggesting a minor role for another RsaA-mediated mechanism.

To further investigate the link between RsaA, the derepression of MgrA, and its subsequent effect on *S. aureus* sensitivity to norfloxacin and cefazolin, we generated a *ΔmgrA* mutant that overproduced RsaA_L_ (*ΔmgrA* pRsaA_L_) and repeated the spot tests (Fig. 4B). These results revealed that the overproduction of RsaA_L_ has no additional effect on the growth of *ΔmgrA* in the presence of norfloxacin, demonstrating that RsaA acts solely through MgrA to mediate its effect on norfloxacin. By contrast, the overproduction of RsaA_L_ led to a slightly increased sensitivity of *ΔmgrA* to cefazolin, indicating that while RsaA mostly acts through MgrA to mediate its effect on cefazolin, another mechanism may be involved.

### The full-length *rsaA* gene is required for expression of the short RsaA form

Having confirmed that the long form of the *rsaA* gene was required for mediating the sensitivity of *S. aureus* to growth in norfloxacin and cefazolin, we wanted to determine why it was required. To investigate this, we first searched for possible open-reading frames in the RsaA_L_ transcript that could translate to a small protein with a regulatory function. This identified one candidate that spanned most of the long-form region; however, mutation of the predicted ORF to block expression had no effect on antibiotic phenotype in spot tests (Fig. S6). Next, we examined whether additional interactions are present between *mgrA* mRNA and RsaA_L_, as compared to what is already known with *mgrA* mRNA and RsaA_S_ (30). However, RNA-RNA interaction prediction software did not find any new interactions present in RsaA_L_ that were not also detected with the short form only (Fig. S7), supporting that for RsaA to pair with *mgrA* mRNA, only its short-form transcript is required.

**Fig 5:**
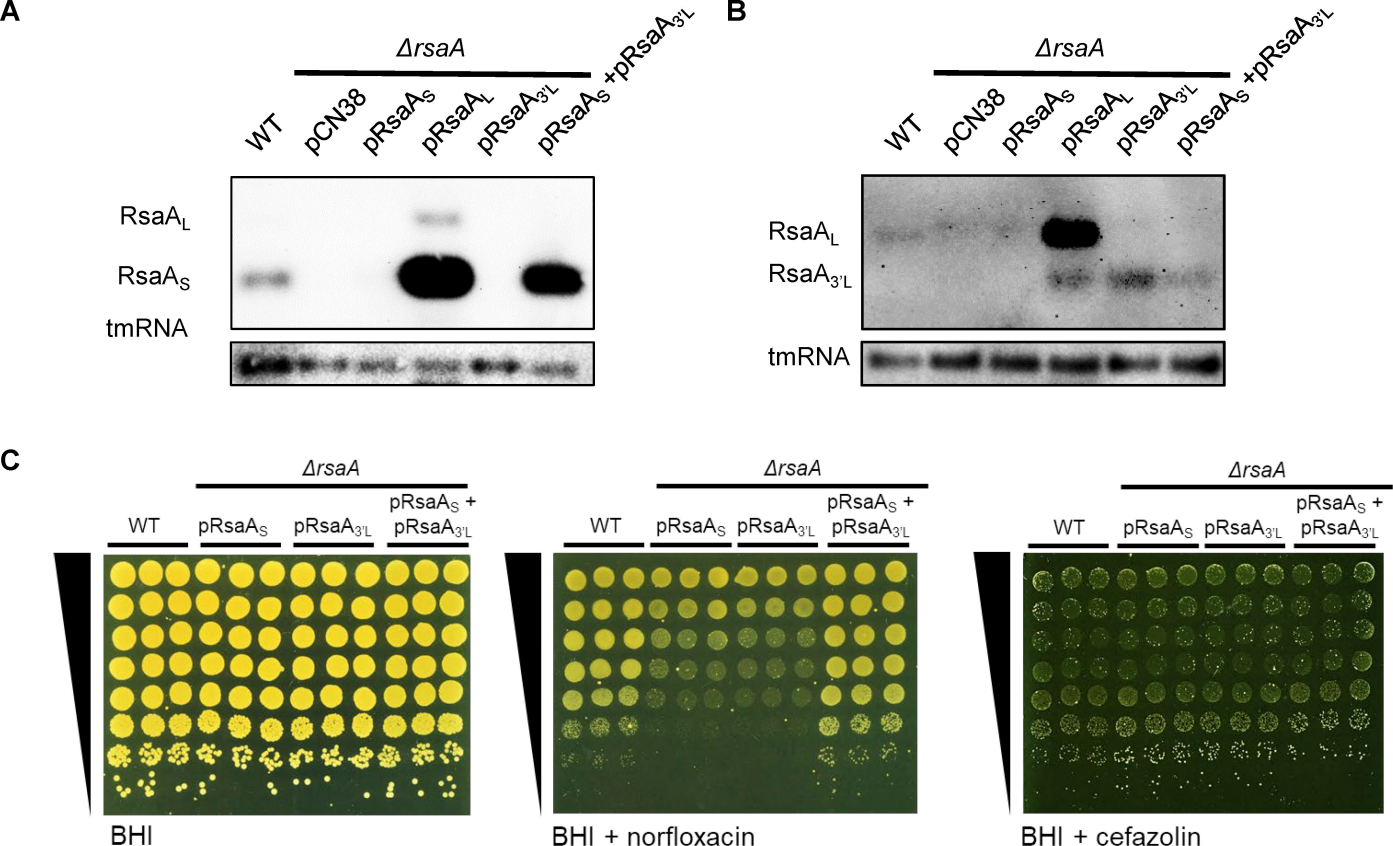
The long RsaA transcript needs to be expressed for the sRNA to function. (A) Northern blot of WT and *ΔrsaA* complemented with empty vector (pCN38), RsaA short form (pRsaA_S_), RsaA long form (pRsaA_L_), RsaA 3′-end of the long form not present in RsaA_S_ name here RsaA_3′L_ (pRsaA_3′L_) and RsaA_S_ + RsaA_3′L_ (pRsaA_S_ + pRsaA_3’L_) sampled at an OD_600_ of 6 and probed for RsaA 5′-end probe to detect both short and long forms. tmRNA was probed as a loading control. Probes are DIG-labeled riboprobes generated from PCR products. A Northern blot with WT and *ΔrsaA* complemented with empty vector, RsaA_S_ and pRsaA_L_, probed for RsaA 5′-end probe using a radioactive labeled DNA probe is presented in Fig. S8A. (B) Northern blot using the same cultures and conditions as for Fig. 5A, probed for RsaA_L_ and RsaA_3′L_ only using an RsaA_L_ 3′-end probe, and tmRNA (loading control). A Northern blot with WT and *ΔrsaA* complemented with empty vector, RsaA_S_ and pRsaA_L_, probed for RsaA_L_ and RsaA_3′L_ only using an RsaA_L_ 3′-end probe using a radioactive labeled DNA probe is presented in Fig. S8B. (C) Growth of WT and *ΔrsaA* complemented with short form (pRsaA_S_), long-region only (pRsaA_3′L_), or both short- and long-form regions (pRsaA_S_ + pRsaA_3′L_), serially diluted 10-fold and spotted onto BHI agar ±0.6 µg/mL norfloxacin or 0.3 µg/mL cefazolin (*n* = 3). BHI, Brain Heart Infusion; WT, wild-type.

Finally, we examined the levels of expression between RsaA_S_ and RsaA_L_. Results showed that there are far higher levels of the short-form transcript in the WT strain when compared to the long-form transcript (Fig. 5A; Fig. S8). This pattern is also reflected in the *ΔrsaA* mutant when complemented with the long form of the gene (Fig. 5A), corresponding with our previous observation that only RsaA_L_ restores the WT phenotype. However, when the *ΔrsaA* mutant was complemented with pRsaA_S_, minimal expression of RsaA_S_ was detected (Fig. 5A; Fig. S8), suggesting that while only the short form is required for sRNA function (Fig. S7), the short form cannot be expressed successfully on its own, and the expression of the long-form transcript is still needed. When we probed the same Northern blot membranes to detect only RsaA_L_ and the long-form region, it was revealed that for the complemented *ΔrsaA* mutant with pRsaA_L_, we could also see a clear band corresponding to the long-form region RsaA_L_, suggesting a cleavage of the long-form region to generate RsaA_S_ (Fig. 5B; Fig. S8).

To investigate the possible contribution of two sRNA fragments in the *rsaA*-dependent phenotype, the *ΔrsaA* mutant was transformed with pRsaA_S_ and pRsaA_3′L_, two plasmids expressing RsaA_S_ and RsaA_3′L_ (RsaA sequence present on RsaA_L_ but not on RsaA_S_), respectively (Fig. S9). Spot tests revealed that although the presence of a single plasmid expressing either RsaA_S_ (via pRsaA_S_) or RsaA_3′L_ (via pRsaA_3′L_) cannot restore the WT phenotype, complementation of *ΔrsaA* was observed when both plasmids were present in the same strain, despite the fact that both fragments (RsaA_S_ and RsaA_3′L_) were not present on the same transcript (Fig. 5C). Northern blot experiments indicate the presence of a single plasmid expressing RsaA_S_ (via pRsaA_S_) leads to a weak quantity of RsaA_S_ likely insufficient to confer a tolerance phenotype. However, the presence of pRsaA_3’L_ expressing RsaA_3′L_ allows the production of an increased quantity of RsaA_S_ restoring the WT phenotype. Surprisingly, a low amount of pRsaA_3′L_ fragment seems sufficient to lead to the accumulation of RsaA_S_ (Fig. 5A and B).

Our data suggest that the RsaA_L_ transcript is possibly cleaved and that the two products are required for RsaA to function as a regulatory RNA.

RNA-RNA interaction prediction software identified an interaction between RsaA_S_ and RsaA_3’L_ (Fig. S10), which suggests that both could be involved in complex formation allowing the accumulation and therefore the activity of RsaA_S_ toward its mRNA targets. Combined, these data demonstrate that the expression of RsaA_L_ is required for the short form to be generated, likely through a cleavage event in which both cleavage products are required to interact for RsaA function.

### RNase Y is not required for *rsaA*-dependent phenotypes

**Fig 6:**
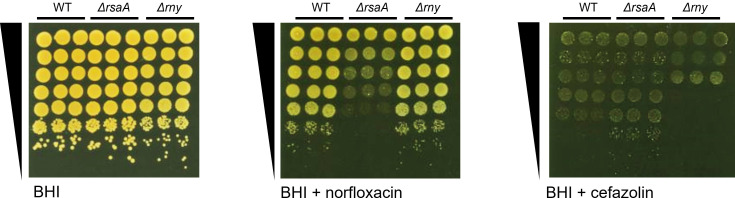
RNase Y does not affect sensitivity to norfloxacin. Growth of WT, *ΔrsaA,* and *Δrny,* serially diluted 10-fold and spotted onto BHI agar ±0.6 µg/mL norfloxacin or 0.3 µg/mL cefazolin (*n* = 3). BHI, Brain Heart Infusion; WT, wild-type.

Experimental and comparative sequence data indicate that at least 15 ribonucleases (RNases) in *S. aureus* are involved in RNA processing and degradation (44). Of these, the main staphylococcal exonucleases are RNase J1/J2 and PNPase, while the main endonucleases are RNase III and RNase Y, both of which influence the stability and processing of RsaA_L_ (29, 45). We sought to assess the effect of RNase III and RNase Y deficiency on the processing of RsaA_L_ into RsaA_S_ and RsaA_3′L_. Attempts to construct an RNase III (*rnc*) mutant in strain HG003 were unsuccessful, potentially due to phage-associated toxin systems, as noted in *Bacillus subtilis* (46). Consequently, only RNase Y was tested. Spot tests comparing plating efficiency on norfloxacin between the WT strain and its RNase Y mutant (*Δrny*) showed no difference, suggesting either that no processing of the long form is necessary for RsaA activity or that RNase Y is not involved in this processing (Fig. 6). However, *Δrny* displayed increased sensitivity to cefazolin compared to either the WT or the *ΔrsaA* mutant, consistent with previous findings that RNase mutants have disordered peptidoglycan layer leading to a hypersensitivity to various antibiotics, including for β-lactam antibiotics like cefazolin, which target the cell wall (47). Since the cefazolin phenotype of *Δrny* was opposite of that observed for *ΔrsaA*, these findings support the conclusion that RNase Y is unlikely to contribute to RsaA activity.

## DISCUSSION

sRNAs play a crucial role in regulating key processes related to antibiotic resistance in gram-negative species (15, 24). These processes include cell envelope modifications (25) and drug efflux pumps (26). However, their impact on antibiotic resistance in gram-positive species has received less attention (15).

In *S. aureus*, the sRNA SprX, also known as RsaOR (20), influences vancomycin and teicoplanin glycopeptide resistance by repressing SpoVG (48). Notably, 6S RNA, which targets RNA polymerase, was shown to contribute to increased growth in low levels of rifampicin and related antibiotics in *S. aureus*, as well as in other distantly related bacteria (28). Antibiotics also influence sRNA gene expression, though connections to antibiotic resistance and/or adaptation are less clear. *S. aureus* sRNAs are differentially expressed after exposure to vancomycin, linezolid, ceftobiprole, and tigecycline (49), and exposure of *S. aureus* to sub-inhibitory concentrations of the β-lactam antibiotic oxacillin enhances bacterial virulence via the sRNA Ssr42 (50).

The data presented here connect the RsaA sRNA to antibiotic resistance mechanisms regarding two antibiotic classes: fluoroquinolones (norfloxacin and ciprofloxacin) and β-lactams (cefazolin, cefalexin, cloxacillin, and penicillin G). Although fluoroquinolones act on DNA replication and β-lactams on the cell wall, mechanisms of resistance to both classes can involve the action of efflux pumps. Our results show that RsaA acts through the master regulator MgrA to mediate the sensitivity of *S. aureus* to antibiotics, linking RsaA with the efflux pumps NorA, NorB, NorC, and AbcA. To our knowledge, RsaA is the first example of sRNA linked to fluoroquinolone resistance mechanisms in *S. aureus*. Of note, in *Escherichia coli*, the sRNA SdsR represses the TolC efflux pump and increases sensitivity to fluoroquinolones (26). SdsR activity is dependent on the RNA chaperone Hfq (26), which is likely not the case for RsaA (51).

We found that the effect of RsaA with fluoroquinolones was fully regulated via MgrA, but this regulation was only partial for β-lactams. Since sRNAs often control the expression of multiple mRNA targets to enable a coordinated regulation of cellular processes (12), it is likely that the RsaA-mediated effect on β-lactam sensitivity is mediated by other mRNA targets. A study investigating the RNA targetome of RsaA identified *ssaA_2* and *ssaA2_3* mRNAs as putative RsaA targets and found that these mRNAs form high-affinity complexes with RsaA *in vitro* (52). Since these two genes belong to the family of staphylococcal secretory antigen A (SsaA) proteins involved in peptidoglycan degradation (53), regulation of these substrates by RsaA may affect changes in sensitivity to β-lactam antibiotics, which function by disrupting peptidoglycan cross-linking during cell wall synthesis (54).

Loss of RsaA was complemented only by the long form of the gene, which demonstrates that the short form of the gene is not sufficient for regulating sensitivity to our tested antibiotics. RsaA_L_ was first detected by Northern blot and believed to originate from read-through at the RsaA_S_ transcriptional terminator (29). Both RsaA_L_ and RsaA_S_ share the same 5′-end, as determined by RACE experiments (21). Our study reveals the importance of the long-form *rsaA_L_* gene being present for the sRNA to function. As no additional interaction sites with *mgrA* mRNA were predicted in RsaA_L_ when compared to RsaA_S_, this correlates with studies on RsaA that have focused only on its short form (29, 30, 52), which is believed to be the functional region. It must be noted that even if RsaA_L_ was not mentioned in these studies, the *ΔrsaA* mutant was complemented using a long stretch of the *S. aureus* genome comprising both *rsaA_L_* and *rsaA_S_* genes and the surrounding genomic region (29, 30, 52).

Our data demonstrate that the long form of the *rsaA* gene is needed for regulating staphylococcal sensitivity to norfloxacin and cefazolin, and that for RsaA to function, the long-form transcript requires processing, most likely through cleavage into two halves. This finding aligns with measurements of the half-lives of RsaA short- and long-form transcripts (29), where RsaA_S_ was found to be >2-fold more stable than RsaA_L_ after treatment with rifampicin to inhibit RNA polymerase (29). Based on experimental and homology data, there are believed to be 15 RNases in *S. aureus* that are involved in RNA processing and/or degradation (44). Of these, the main staphylococcal RNases are the exonucleases RNase J1/J2 and PNPase, and the endonucleases RNase III and RNase Y, the latter two of which have been reported to affect the stability and processing of RsaA_L_ (29, 45). We found that RNase Y is unlikely to be the processing enzyme for the cleavage of RsaA_L_ into two halves, though the lack of a viable RNase III mutant in HG003 does not exclude this enzyme from this role.

In *S. aureus*, RsaE is a sRNA possibly processed from a longer transcript to interact more efficiently with some targets, thereby expanding the range of recognised targets. However, this feature is, so far, only reported for its *B. subtilis* and *Staphylococcus epidermidis* orthologs (55, 56). In the case of RsaA, we were able to restore the WT phenotype in a *ΔrsaA* mutant by expressing both RsaA_S_ and the long region of RsaA_L_ from different plasmids (pRsaA_S_ and pRsaA_3′L_), such that the RsaA_L_ long region is most likely interacting with the short form of RsaA to enable its function, possibly by stabilising the sRNA and/or by promoting interactions with *mgrA* mRNA. We found that the predicted interaction site of RsaA_S_ with the RsaA_L_ long region has minimal overlap with its interaction site with *mgrA* mRNA, suggesting that both interactions could occur at the same time. Complexes forming between RsaA and other mRNAs/sRNAs have been detected by RNase III CLASH, including with *mgrA* mRNA and RNAIII sRNA, both of which interact with the short-form region of RsaA (57). Although an RNase III complex with RsaA_S_ and RsaA_3’L_ was not mentioned, hybrids with high sequence similarity were excluded, and it is possible that the similar sRNA-sRNA interaction may not have been considered.

Additionally, the RsaA_L_ long region was unexpectedly lower in abundance than the short form of RsaA despite being required for its function, suggesting that any interactions may be transient and/or that RsaA_3′L_ may be more susceptible to degradation. We also observed that the abundance of the RsaA_L_ long region from pRsaA_3′L_ was much higher when compared to the WT, where it was not detectable. It is possible that these higher levels were due to RsaA_L_ being supplied in multicopy form via a plasmid in the *ΔrsaA* pRsaA_L_ strain, which is reflected in the much lower abundance of RsaA_L_ in the WT strain when compared to the *ΔrsaA* mutant expressing pRsaA_L_.

sRNAs control many cellular processes and enable rapid adaptation to stress. Here, we show that staphylococcal RsaA acts via the master regulator MgrA to mediate sensitivity to two different antibiotic classes, fluoroquinolones and β-lactams. In addition, we revealed that this regulation requires the full-length version of the *rsaA* gene, the transcript of which is likely processed by the cell to produce two halves that interact to produce functional RsaA. Therefore, exposure of *S. aureus* to sub-inhibitory fluoroquinolone or β-lactam concentrations can alter the expression of their corresponding efflux pumps, adapting the bacterium to growth in the presence of antibiotics.

## MATERIALS AND METHODS

### Bacterial strains, plasmids, and growth conditions

The bacterial strains used in this study are listed in Table S1. Experiments were performed with HG003, a *S. aureus* model strain widely used for regulation studies (35). Plasmids were engineered by Gibson assembly (58) in *E. coli* IM08B (59) as described (Table S2), using the indicated appropriate primers (Table S3) for PCR amplification. Plasmids were verified by DNA sequencing and transformed into HG003 or its derivatives, as appropriate. HG003 *ΔmgrA* and *ΔrsaA ΔmgrA* double mutants were constructed by transferring *ΔmgrA*::tetM (60) into HG003 and HG003 *ΔrsaA*::tag075 using phage-mediated transduction.

*S. aureus* was routinely cultured in Brain Heart Infusion (BHI) broth at 37°C, with shaking (180 rpm). *E. coli* was grown in Lysogeny Broth at 37°C with shaking (180 rpm). Media were supplemented with antibiotics as required: ampicillin 100 µg/mL for *E. coli*; chloramphenicol 5 µg/mL, tetracycline 1 µg/mL, and kanamycin 90 µg/mL for *S. aureus*.

### Fitness competition assay

Mutants with altered fitness in the presence of norfloxacin were identified and analzed using a reported strategy (27) with three independent libraries containing 48 mutants (Table S4). The libraries were grown at 37°C in BHI or BHI + 0.25 µg/mL norfloxacin for 3 days. Overnight cultures were diluted 1,000 times into fresh medium for each day. Samples were withdrawn at an OD_600_ of 1 and after overnight growth as indicated (Fig. 1A).

### Spot tests

To assess mutant phenotypes, bacterial growth in the presence of antibiotics was visualized by spot tests, which enable subtle growth phenotypes to be detected, such as with sRNA mutants. A total of 5 µL of 10-fold serial dilutions of overnight cultures (from neat to 10^−7^ dilutions) were spotted on agar plates containing either BHI or BHI + antibiotic at the indicated concentrations. Agar plates were incubated for 24 h at 37°C.

### Measurement of bacterial growth in liquid culture

To measure growth of *S. aureus* in liquid culture, bacteria were first grown to stationary phase in BHI at 37°C (180 rpm), then inoculated 1/50 into a flat-bottomed 96-well plate (200 µL total volume) and placed into a CLARIOstar Omega plate reader (BMG Labtech). Antibiotics were added to the media as needed, at the indicated concentrations. Bacteria were grown for 20 h at 37°C (300 rpm), and absorbance at 600 nm was measured every 30 min.

### Northern blots

Total RNA preparations and Northern blots were performed as previously described (61). A total of 20 µg of total RNA samples was separated either by agarose (1.3%) gel electrophoresis. Membranes were probed with either primer (Table S3) ^32^P-labeled using Terminal Deoxynucleotidyl Transferase (ThermoScientific) and scanned using an Amersham Typhoon imager or T7 generated riboprobes (for PCR matrices, see Table S3) DIG-labeled using the DIG Northern Starter Kit (Sigma-Aldrich) according to the manufacturer’s instructions and scanned using a Bio-Rad ChemiDoc imager.

## SUPPLEMENTAL MATERIAL

Supplemental material aac.01176-24-s0001.pdf Tables S1 to S5; Fig. S1 to S10.

ASM does not own the copyrights to Supplemental Material that may be linked to, or accessed through, an article. The authors have granted ASM a non-exclusive, world-wide license to publish the Supplemental Material files. Please contact the corresponding author directly for reuse.
