# Supplementary material for: A small regulatory RNA controls antibiotic adaptation in Staphylococcus aureus by modulating efflux pump expression
Source: Antimicrob Agents Chemother. 2025 Apr 3;69(5):e01176-24. doi: 10.1128/aac.01176-24 (PMC12057360; doi:10.1128/aac.01176-24)
Supplement: Supplemental material — Tables S1 to S5; Fig. S1 to S10. [file aac.01176-24-s0001.pdf]

Supplementary information file

A small regulatory RNA controls antibiotic adaptation in *Staphylococcus aureus* by modulating efflux pump expression

Kam Pou Ha, Etornam Kofi Kumeko, Philippe Bouloc#

Université Paris-Saclay, CEA, CNRS, Institute for Integrative Biology of the Cell (I2BC), 91198, Gif-sur-Yvette, France.

#For correspondence: philippe.bouloc@i2bc.paris-saclay.fr

Table S1. *Staphylococcus aureus* strains 2

Table S2. Plasmids 6

Table S3. Primers 7

Table S4. Composition of libraries for fitness competition assays 8

Table S5. Minimum Inhibitory Concentration (MIC) of norfloxacin in *S. aureus* HG003 strains. 10

Figure S1. Growth of WT and *ΔrsaA* in 0.25 µg/ml norfloxacin. 11

Figure S2. Predicted secondary structure of $RsaA_L$ 12

Figure S3. Growth of WT and *rsaA* strains in BHI only. 13

Figure S4. Growth of WT and *rsaA* strains in vancomycin. 14

Figure S5. Spot tests of *mgrA* mutants grown on BHI agar alone. 15

Figure S6. RsaA-mediated sensitivity to norfloxacin and cefazolin is not caused by protein expression from a potential open-reading frame within $RsaA_L$ 16

Figure S7. Predicted RNA-RNA interactions between *mgrA* mRNA and $RsaA_L$ all fall within the first 144 bp of the $RsaA_L$ transcript, which corresponds to the short form region 17

Figure S8. Accumulation of $RsaA_S$ in presence of $RsaA_L$. 18

Figure S9. Simplified diagram of RsaA secondary structure with proposed cleavage site. 19

Figure S10. Predicted RNA-RNA interaction between $RsaA_S$ and $RsaA_{3'L}$ 20

References 21

Table S1. ***Staphylococcus aureus*** **strains**

**Name**	**Genotype**	**Source**
HG003	NCTC8325 *rsbU* and *tcaR* repaired	(1)
**Tagged sRNA mutants used in the fitness competition assay**		
SAPhB618	HG003 Δ*rnaIII*::tag004	(2)
SAPhB347	HG003 Δ*rsaOG*::tag009	(2)
SAPhB349	HG003 Δ*rsaG*::tag011	(2)
SAPhB368	HG003 Δ*teg147*::tag018	(2)
SAPhB380	HG003 Δ*rsaB*::tag025	(2)
SAPhB682	HG003 Δ*rsaD*::tag026	(2)
SAPhB386	HG003 Δ*teg116*::tag030	(2)
SAPhB397	HG003 Δ*sau85*::tag038	(2)
SAPhB402	HG003 Δ*sau6353*::tag042	(2)
SAPhB404	HG003 Δ*rsaE*::tag045	(2)
SAPhB412	HG003 Δ*ssr42*::tag050	(2)
SAPhB415	HG003 Δ*teg155*::tag053	(2)
SAPhB960	HG003 Δ*sprF3*::tag070	(3)
SAPhB961	HG003 Δ*sprF3*::tag070	(3)
SAPhB962	HG003 Δ*sprF3*::tag070	(3)
SAPhB862	HG003 Δ*sRNA334*::tag073	(3)
SAPhB863	HG003 Δ*sRNA334*::tag073	(3)
SAPhB864	HG003 Δ*sRNA334*::tag073	(3)
SAPhB943	HG003 Δ*rsaA*::tag075	(3)
SAPhB944	HG003 Δ*rsaA*::tag075	(3)
SAPhB945	HG003 Δ*rsaA*::tag075	(3)
SAPhB890	HG003 Δ*sau76*::tag076	(3)
SAPhB891	HG003 Δ*sau76*::tag076	(3)
SAPhB962	HG003 Δ*sau76*::tag076	(3)
SAPhB883	HG003 Δ*rsaOI*::tag077	(3)
SAPhB884	HG003 Δ*rsaOI*::tag077	(3)
SAPhB885	HG003 Δ*rsaOI*::tag077	(3)
SAPhB865	HG003 Δ*teg16*::tag080	(3)
SAPhB866	HG003 Δ*teg16*::tag080	(3)
SAPhB867	HG003 Δ*teg16*::tag080	(3)
SAPhB871	HG003 Δ*sRNA287*::tag085	(3)
SAPhB872	HG003 Δ*sRNA287*::tag085	(3)
SAPhB873	HG003 Δ*sRNA287*::tag085	(3)
SAPhB874	HG003 Δ*sRNA71*::tag086	(3)

SAPhB875	HG003 *ΔsRNA71*::tag086	(3)
SAPhB876	HG003 *ΔsRNA71*::tag086	(3)
SAPhB907	HG003 *ΔsRNA209*::tag093	(3)
SAPhB908	HG003 *ΔsRNA209*::tag093	(3)
SAPhB909	HG003 *ΔsRNA209*::tag093	(3)
SAPhB899	HG003 *Δteg106*::tag095	(3)
SAPhB900	HG003 *Δteg106*::tag095	(3)
SAPhB946	HG003 *Δteg106*::tag095	(3)
SAPhB921	HG003 *ΔsRNA260*::tag096	(3)
SAPhB922	HG003 *ΔsRNA260*::tag096	(3)
SAPhB947	HG003 *ΔsRNA260*::tag096	(3)
SAPhB910	HG003 *ΔsRNA345*::tag097	(3)
SAPhB911	HG003 *ΔsRNA345*::tag097	(3)
SAPhB912	HG003 *ΔsRNA345*::tag097	(3)
SAPhB932	HG003 *ΔncRNA2*::tag099	(3)
SAPhB933	HG003 *ΔncRNA2*::tag099	(3)
SAPhB934	HG003 *ΔncRNA2*::tag099	(3)
SAPhB940	HG003 *ΔncRNA3*::tag100	(3)
SAPhB941	HG003 *ΔncRNA3*::tag100	(3)
SAPhB942	HG003 *ΔncRNA3*::tag100	(3)
SAPhB954	HG003 *ΔssrS*::tag107	(3)
SAPhB955	HG003 *ΔssrS*::tag107	(3)
SAPhB956	HG003 *ΔssrS*::tag107	(3)
SAPhB1006	HG003 *ΔsprF1*::tag110	(3)
SAPhB1007	HG003 *ΔsprF1*::tag110	(3)
SAPhB1008	HG003 *ΔsprF1*::tag110	(3)
SAPhB974	HG003 *ΔsprX2*::tag111	(3)
SAPhB975	HG003 *ΔsprX2*::tag111	(3)
SAPhB997	HG003 *ΔsprX2*::tag111	(3)
SAPhB978	HG003 *ΔsprY2*::tag112	(3)
SAPhB979	HG003 *ΔsprY2*::tag112	(3)
SAPhB980	HG003 *ΔsprY2*::tag112	(3)
SAPhB957	HG003 *ΔsprY3*::tag113	(3)
SAPhB958	HG003 *ΔsprY3*::tag113	(3)
SAPhB959	HG003 *ΔsprY3*::tag113	(3)
SAPhB901	HG003 *Δsau41*::tag115	(3)
SAPhB902	HG003 *Δsau41*::tag115	(3)
SAPhB903	HG003 *Δsau41*::tag115	(3)
SAPhB948	HG003 *Δsau5949*::tag117	(3)
SAPhB949	HG003 *Δsau5949*::tag117	(3)
SAPhB950	HG003 *Δsau5949*::tag117	(3)
SAPhB966	HG003 *ΔsprF2*::tag118	(3)

SAPhB967	HG003 Δ*sprF2*::tag118	(3)
SAPhB998	HG003 Δ*sprF2*::tag118	(3)
SAPhB1031	HG003 Δ*sprB*::tag121	(3)
SAPhB1032	HG003 Δ*sprB*::tag121	(3)
SAPhB1033	HG003 Δ*sprB*::tag121	(3)
SAPhB1242	HG003 Δ*rsaC*::tag133	(3)
SAPhB1243	HG003 Δ*rsaC*::tag133	(3)
SAPhB1244	HG003 Δ*rsaC*::tag133	(3)
SAPhB1234	HG003 Δ*S204*::tag134	(3)
SAPhB1235	HG003 Δ*S204*::tag134	(3)
SAPhB1236	HG003 Δ*S204*::tag134	(3)
SAPhB1231	HG003 Δ*isrR*::tag135	(3)
SAPhB1232	HG003 Δ*isrR*::tag135	(3)
SAPhB1233	HG003 Δ*isrR*::tag135	(3)
SAPhB1239	HG003 Δ*S808*::tag137	(3)
SAPhB1240	HG003 Δ*S808*::tag137	(3)
SAPhB1241	HG003 Δ*S808*::tag137	(3)
SAPhB1015	*locus3*::tag139	(3)
SAPhB1016	*locus3*::tag139	(3)
SAPhB1017	*locus3*::tag139	(3)
SAPhB1012	*locus2*::tag140	(3)
SAPhB1013	*locus2*::tag140	(3)
SAPhB1014	*locus2*::tag140	(3)
SAPhB1009	*locus1*::tag141	(3)
SAPhB1010	*locus1*::tag141	(3)
SAPhB1011	*locus1*::tag141	(3)
SAPhB1018	HG003 Δ*sau5971*::tag142	(3)
SAPhB1019	HG003 Δ*sau5971*::tag142	(3)
SAPhB1020	HG003 Δ*sau5971*::tag142	(3)
SAPhB976	HG003 Δ*sprA1*::tag144	(3)
SAPhB977	HG003 Δ*sprA1*::tag144	(3)
SAPhB996	HG003 Δ*sprA1*::tag144	(3)
SAPhB1027	HG003 Δ*sprX2*::tag145	(3)
SAPhB1028	HG003 Δ*sprX2*::tag145	(3)
SAPhB1029	HG003 Δ*sprX2*::tag145	(3)
SAPhB1003	HG003 Δ*sprX1*::tag146	(3)
SAPhB1004	HG003 Δ*sprX1*::tag146	(3)
SAPhB1005	HG003 Δ*sprX1*::tag146	(3)
SAPhB971	HG003 Δ*rsaH*::tag147	(3)
SAPhB972	HG003 Δ*rsaH*::tag147	(3)
SAPhB973	HG003 Δ*rsaH*::tag147	(3)
SAPhB1021	HG003 Δ*sprY1*::tag148	(3)

SAPhB1022	HG003 Δ*sprY1*::tag148	(3)
SAPhB1023	HG003 Δ*sprY1*::tag148	(3)

**RsaA complementation**		
SAPhB2027	HG003 Δ*rsaA*::tag075 pCN38	SAPhB943 + pCN38
SAPhB2029	HG003 Δ*rsaA*::tag075 pRsaA$_S$	SAPhB943 + pRsaA$_S$
SAPhB2047	HG003 Δ*rsaA*::tag075 pRsaA$_L$	SAPhB943 + pRsaA$_L$
SAPhB2342	HG003 Δ*rsaA*::tag075 pRsaA$_{3'L}$	SAPhB943 + pRsaA$_{3'L}$
SAPhB2344	HG003 Δ*rsaA*::tag075 pRsaA$_S$ pRsaA$_{3'L}$	SAPhB2029 + pRsaA$_{3'L}$
SAPhB2183	HG003 Δ*rsaA* pRsaA$_L$-Stop	SAPhB943 + pRsaA$_L$-Stop

***mgrA* mutants**		
AH3455	USA300 LAC* Δ*mgrA*::tetM	(4)
SAPhB2135	HG003 Δ*mgrA*::tetM	HG003 + ϕ80 lysate from AH3455
SAPhB2137	HG003 Δ*rsaA*::tag075 Δ*mgrA*::tetM	SAPhB943 + ϕ80 lysate from AH3455
SAPhB2160	HG003 Δ*mgrA*::tetM pCN38	SAPhB2135 + pCN38
SAPhB2162	HG003 Δ*mgrA*::tetM pRsaA$_L$	SAPhB2135 + pRsaA$_L$

***rny* mutant**		
SAPhB442	HG003 Δ*rny*	Laboratory collection

Table S2. Plasmids

Name	Description	Source
pCN38	Shuttle vector, pT181 replicon, Cm^R	(5)
pRsaA$_S$	*rsaA$_S$* under the control of its endogenous promoter. RsaA nucleotide positions 1-144 (Figure S2) were cloned into pCN38.	1489/1490 on pCN38; 1469/1470 on HG003
pRsaA$_L$	*rsaA$_L$* under the control of its endogenous promoter	1489/1490 on pCN38; 2898/2899 on HG003
pRsaA$_L$-Stop	*rsaA$_L$* under the control of its endogenous promoter (as pRsaA$_L$), with a point mutation (ATG→TAG) to block expression of a predicted ORF	2898/3020 and 2899/3021 on HG003, then joined using 2898/2899
pCN34	Shuttle vector, pT181 replicon, Km^R	(5)
pRsaA$_{3'L}$	*rsaA$_{3'L}$* under the control of its endogenous promoter. RsaA nucleotide positions 145-282 (Figure S2) were cloned into pCN34.	1489/1490 on pCN34; 3212/3213 and 3214/3215 on HG003

Table S3. Primers

Name	Description	Sequence 5'-3'
1489	pCN38/pCN34_F	CAGTTGCGCAGCCTGAATGG
1490	pCN38/pCN34_R	CCTCTAGAGTCGACCTGCAG
1469	RsaA-S_F	CTGCAGGTCGACTCTAGAGGTGTCCATTTCATCACCATTGTTT
1470	RsaA-S_R	CCATTCAGGCTGCGCAACTGCCGTATACATAAGACGTGATTTGG
2898	RsaA-L_F	GGTCGACTCTAGAGGCGGAATTTTTAGTAGTTAAAATTCTCTTG
2899	RsaA-L_R	CAGGCTGCGCAACTGTTTATAAATTAATAAAAAAATTCCAAGCTTATC
3212	PrsaA_F	CATGCCTGCAGGTCGACTCTAGAGGCGGAATTTTTAGTAGTTAAAATT CTCTTG
3213	PrsaA_R	AATGTATTATCAAATATTATTTTAACCTATTTGAAATATTTGAAAC
3214	rsaA-3'L_F	TTAAAATAATATTTGATAATACATTAGCACATATATATAAGTTTAAAC
3215	rsaA-3'L_R	ATTCGCCATTCAGGCTGCGCAACTGTTTATAAATTAATAAAAAAATTCC AAGCTTATC
3020	rsaA-L-STOP_R	ACTTTGCTCTAAGCAAAGTGTACCCGAG
3021	rsaA-L-STOP_F	CTTTGCTTAGAGCAAAGTGTACTTTGTTATTGATAATAC
**Northern blot (radioactive probes)**		
3137	RsaA 5'-end	ACAGTCGCTACTCTATACAATTTTTGTAATGGTTAACT
3211	$RsaA_L$ 3'-end	TCCCGTATACATAAGACGTGATTTGGTAAATAGTTG
2627	tmRNA	CTTCAAACGGCAGTGTTTAGC
**Northern blot (to generate DIG-labelled riboprobes)**		
3408	T7-anti$RsaA_L$_F	TAATACGACTCACTATAGGGCTTATCGGTTTAAGCTTGGAA
3409	antiRsaAL_R	GTTATTGATAATACATTAGCACATATAT
3410	T7-anti$RsaA_S$_F	TAATACGACTCACTATAGGGCTCATAGCAAAGTGTACCC
3411	anti$RsaA_S$_R	GTTAACCATTACAAAAATTGTATAGAGTAG

Table S4. Composition of libraries for fitness competition assays

Mutant	Library 1	Library 2	Library 3
rnaIII::tag004	SAPhB618	SAPhB618	SAPhB618
rsaOG::tag009	SAPhB347	SAPhB347	SAPhB347
rsaG::tag011	SAPhB349	SAPhB349	SAPhB349
teg147::tag018	SAPhB368	SAPhB368	SAPhB368
rsaB::tag025	SAPhB380	SAPhB380	SAPhB380
rsaD::tag026	SAPhB682	SAPhB682	SAPhB682
teg116::tag030	SAPhB386	SAPhB386	SAPhB386
sau85::tag038	SAPhB397	SAPhB397	SAPhB397
sau6353::tag042	SAPhB402	SAPhB402	SAPhB402
rsaE::tag045	SAPhB404	SAPhB404	SAPhB404
ssr42::tag050	SAPhB412	SAPhB412	SAPhB412
teg155::tag053	SAPhB415	SAPhB415	SAPhB415
sprF3::tag070	SAPhB960	SAPhB961	SAPhB962
sRNA334::tag073	SAPhB862	SAPhB863	SAPhB864
rsaA::tag075	SAPhB943	SAPhB944	SAPhB945
sau76::tag076	SAPhB890	SAPhB891	SAPhB962
rsaOI::tag077	SAPhB883	SAPhB884	SAPhB885
teg16::tag080	SAPhB865	SAPhB866	SAPhB867
sRNA287::tag085	SAPhB871	SAPhB872	SAPhB873
sRNA71::tag086	SAPhB874	SAPhB875	SAPhB876
sRNA209::tag093	SAPhB907	SAPhB908	SAPhB909
teg106::tag095	SAPhB899	SAPhB900	SAPhB901
sRNA260::tag096	SAPhB921	SAPhB922	SAPhB947
sRNA345::tag097	SAPhB910	SAPhB911	SAPhB912
ncRNA2::tag099	SAPhB932	SAPhB933	SAPhB934
ncRNA3::tag100	SAPhB940	SAPhB941	SAPhB942
ssrS::tag107	SAPhB954	SAPhB955	SAPhB956
sprF1::tag110	SAPhB1006	SAPhB1007	SAPhB1008
sprX2::tag111	SAPhB974	SAPhB975	SAPhB976
sprY2::tag112	SAPhB978	SAPhB979	SAPhB980
sprY3::tag113	SAPhB957	SAPhB958	SAPhB959
sau41::Tag115	SAPhB901	SAPhB902	SAPhB903
sau5949::tag117	SAPhB948	SAPhB949	SAPhB950
sprF2::tag118	SAPhB966	SAPhB967	SAPhB998
sprB::tag121	SAPhB1031	SAPhB1032	SAPhB1033
rsaC::tag133	SAPhB1242	SAPhB1243	SAPhB1244
S204::tag134	SAPhB1234	SAPhB1235	SAPhB1236
S596::tag135	SAPhB1231	SAPhB1232	SAPhB1233
S808::tag137	SAPhB1239	SAPhB1240	SAPhB1241

locus3::tag139	SAPhB1015	SAPhB1016	SAPhB1017
locus2::tag140	SAPhB1012	SAPhB1013	SAPhB1014
locus1::tag141	SAPhB1009	SAPhB1010	SAPhB1011
sau5971::tag142	SAPhB1018	SAPhB1019	SAPhB1020
sprA1::tag144	SAPhB976	SAPhB977	SAPhB996
sprX2::tag145/*sprX1*::tag149	SAPhB1027	SAPhB1028	SAPhB1029
sprX1::tag146	SAPhB1003	SAPhB1004	SAPhB1005
rsaH::tag147	SAPhB971	SAPhB972	SAPhB973
sprY1::tag148	SAPhB1021	SAPhB1022	SAPhB1023

Table S5. Minimum Inhibitory Concentration (MIC) of norfloxacin in *S. aureus* HG003 strains.

Strain	MIC (µg/ml) of norfloxacin
WT	0.5
ΔrsaA	0.5

MICs were determined using a serial broth dilution protocol as described (6). Bacteria were diluted to 1 x 10^5 CFU/ml and incubated in flat-bottomed 96-well plates with a range of antibiotic concentrations for 17 h at 37 °C under static conditions. Results are recorded as the lowest concentration of antibiotic at which no visible growth was observed (n ≥ 3; median MIC as shown)

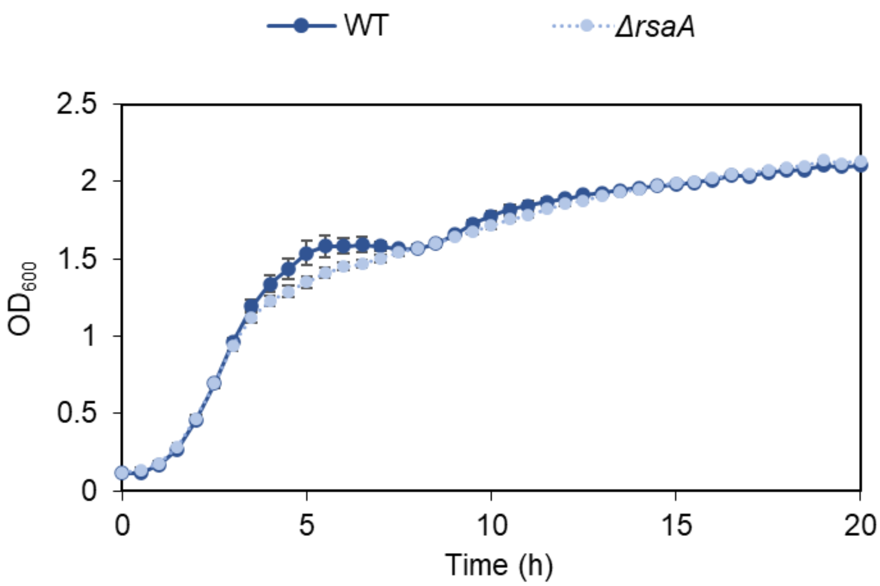

Figure S1. Growth of WT and *ΔrsaA* in 0.25 µg/ml norfloxacin.
Strains were grown in BHI broth supplemented with norfloxacin at the concentrations indicated (n = 3). Error bars represent the standard deviation of the mean.

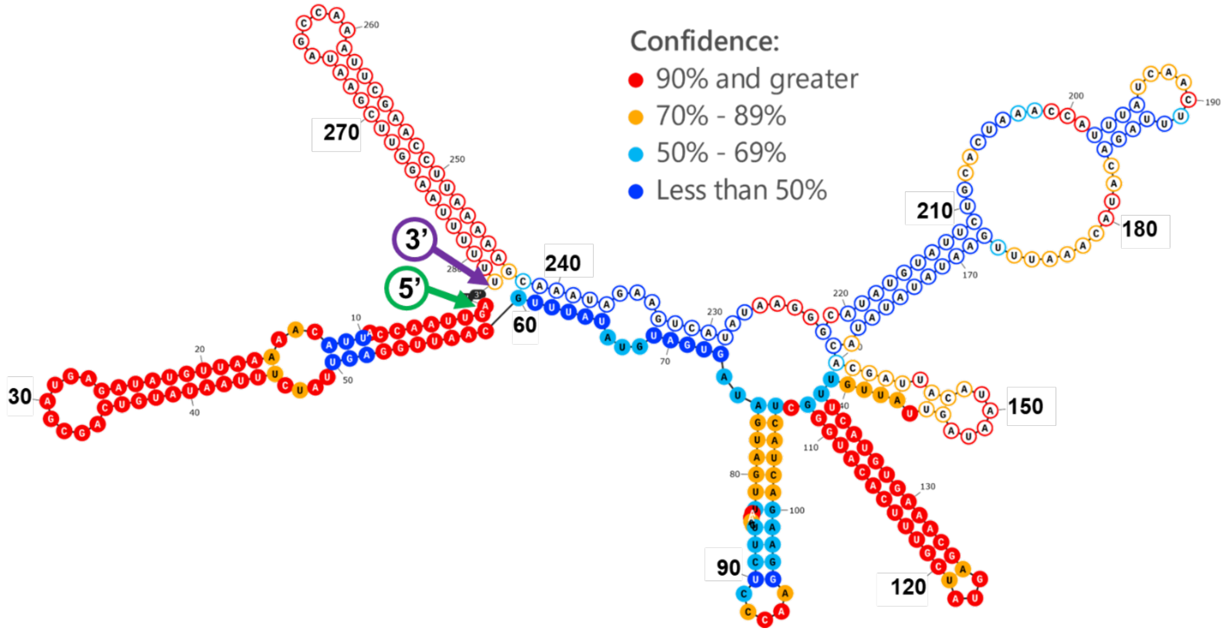

Figure S2. Predicted secondary structure of RsaA$_L$.
Secondary structure of RsaA long form (282 bp), as predicted by SnapGene software (version 7.0.3). The region with filled circles refers to the structure of the RsaA short form region (1-144 bp), which has been experimentally confirmed as shown (7). 5'- and 3'-ends are indicated by green and purple arrows, respectively. Nucleotide positions 1-144 were cloned into pCN38-rsaA$_S$ to express the short-form region of RsaA. Numbers in black refer to the base position within RsaA$_L$. Prediction confidence is indicated by colour, as described in the legend. Image generated using SnapGene software (from Dotmatics; available at snapgene.com).

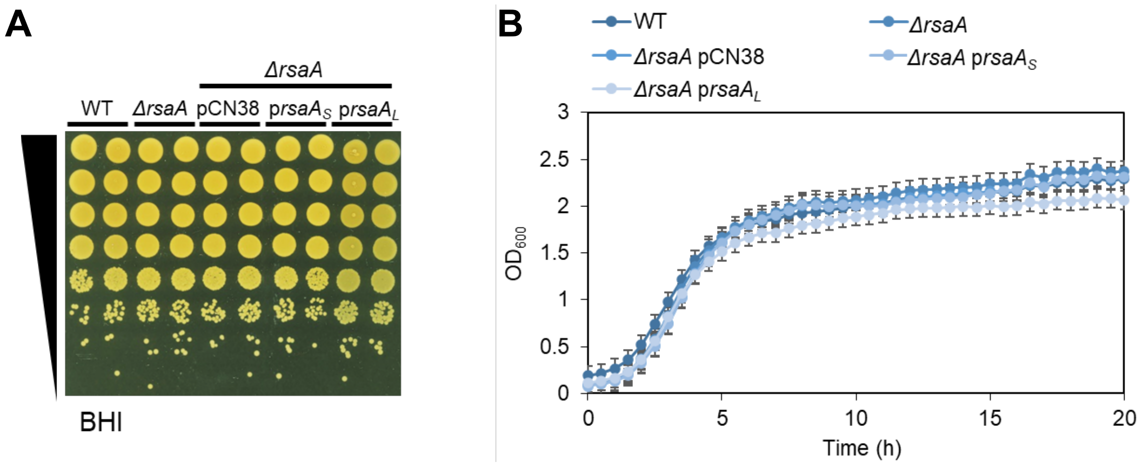

Figure S3. Growth of WT and *rsaA* strains in BHI only.
Growth of WT, *rsaA* mutant, empty vector (pCN38) and mutants complemented with RsaA short form (pRsaA$_S$) or long form (pRsaA$_L$), either **(A)** serially diluted 10-fold and spotted onto BHI agar (n = 3), or **(B)** grown in BHI broth (n = 3). Error bars represent the standard deviation of the mean.

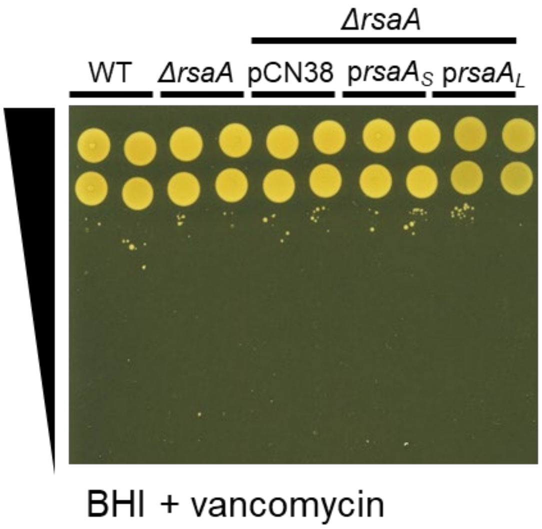

Figure S4. Growth of WT and *rsaA* strains in vancomycin.
Growth of WT, *rsaA* mutant, empty vector (pCN38) and mutants complemented with RsaA short form (pRsaA$_S$) or long form (pRsaA$_L$), serially diluted 10-fold and spotted onto BHI agar + 2 µg/ml vancomycin (n = 3). Spot tests in BHI broth alone are shown in Figure S2.

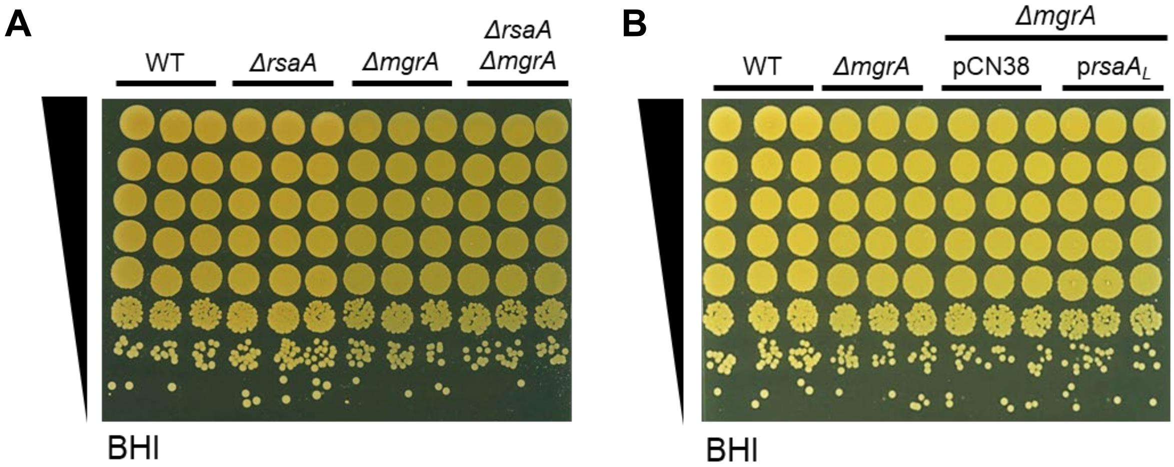

Figure S5. Spot tests of *mgrA* mutants grown on BHI agar alone.
(A) Growth of WT, *ΔrsaA*, *ΔmgrA* and *ΔrsaA ΔmgrA* strains, serially diluted 10-fold and spotted onto BHI agar (n = 3). **(B)** Growth of WT, *ΔmgrA* and *ΔmgrA* complemented with empty vector (pCN38) or long form (pRsaA$_L$), serially diluted 10-fold and spotted onto BHI agar (n = 3).

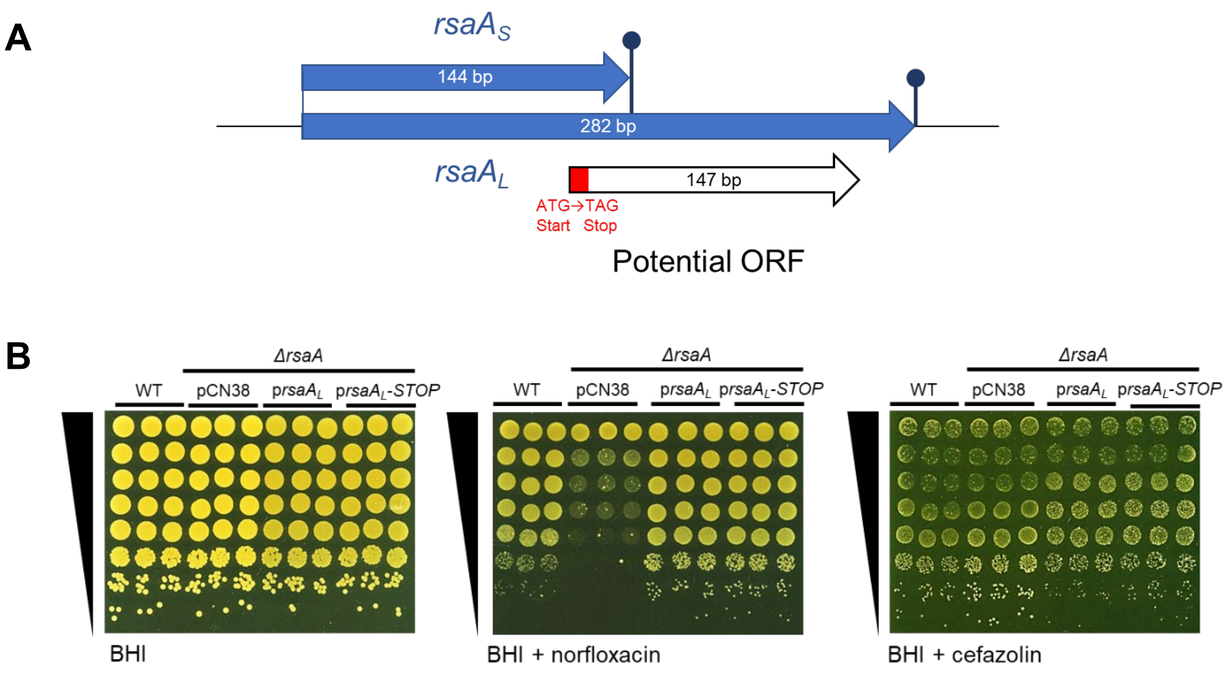

Figure S6. RsaA-mediated sensitivity to norfloxacin and cefazolin is not caused by protein expression from a potential open-reading frame within RsaA$_L$

(A) Schematic showing a potential open-reading frame (ORF) covering the long region of RsaA, as predicted by the NCBI ORF Finder(8). Arrows represent genes/ORFs, dark blue sticks represent transcription terminators. The red box represents the introduction of a nonsense mutation where the start codon (ATG) is replaced by a stop codon (TAG). NCBI ORF Finder search criteria were set to all start codons ("ATG" and "alternative") with a minimal ORF length of 30nt. **(B)** Mutation of the predicted ORF to block expression has no effect on the RsaA-mediated sensitivity to norfloxacin or cefazolin. Growth of WT and *ΔrsaA* complemented with empty vector (pCN38), long form (pRsaA$_L$), or long form with a nonsense mutation of a predicted ORF (pRsaA$_L$-Stop), serially diluted 10-fold and spotted onto BHI agar +/- 0.6 µg/ml norfloxacin or 0.3 µg/ml cefazolin (n = 3).

```
                 Predicted interaction 1

                          -11                     11
                            |                      |
mgrA      5'-    ..CUGGA              UGU   G     ACAUA..    -3'
mRNA                    GGAGAACUUUAA   CU  AUCA
                        ||||||||||||   ||  ||||
RsaAL                   CCUCUUGAAAUU   GA  UAGU
sRNA      3'-UUU..GGAAC             GAU         GAUGU..UGA-5'
                            |                      |
                          92                      72

          Energy: -9.84 kcal/mol
          Hybridisation Energy: -19.54 kcal/mol
```

```
                 Predicted interaction 2

                           27                          51
                            |                           |
mgrA      5'-    ..AAAGA     CUAU    UUAG               GCUCA..    -3'
mRNA                    ACAG    GCU     UUUGUACAAU
                        ||||    |||     ||||||||||
RsaAL                   UGUC    CGA     AGAUAUGUUA
sRNA      3'-UUU..UAAUA     AG      UG                  AAAAC..UGA-5'
                            |                           |
                           38                          18

          Energy: -3.91 kcal/mol
          Hybridisation Energy: -14.08 kcal/mol
```

```
                 Predicted interaction 3

                           81         89
                            |         |
mgrA      5'-UGA..UACUC              UUUUA..AGU-3'
mRNA                    UAACAAAGU
                        |||||||||
RsaAL                   AUUGUUUCA
sRNA      3'-UUU..UAGUU              UGUGA..UGA-5'
                            |         |
                          143        135

          Energy: -2.63 kcal/mol
          Hybridisation Energy: -8.37 kcal/mol
```

Figure S7. Predicted RNA-RNA interactions between *mgrA* mRNA and $RsaA_L$ all fall within the first 144 bp of the $RsaA_L$ transcript, which corresponds to the short form region

Predicted interaction between $RsaA_L$ (282bp) and *mgrA* mRNA, as proposed by IntaRNA RNA-RNA interaction prediction software (9). The first two predicted interactions correspond to the equivalent confirmed interactions in $RsaA_S$ (7). The third interaction is also predicted when $RsaA_S$ is input into IntaRNA with *mgrA* mRNA. The short form of RsaA covers base positions 1-144 of the long-form RsaA transcript, $RsaA_L$ (282 bp). Input of *mgrA* mRNA was +/- 100nt of the start codon (green). SD, Shine-Dalgarno sequence (blue). The energy score (bold) of a predicted RNA-RNA interaction is the sum of the hybridisation free energy of the interacting sub-sequences, and the free energies required to unfold the interaction sites in both RNA molecules.

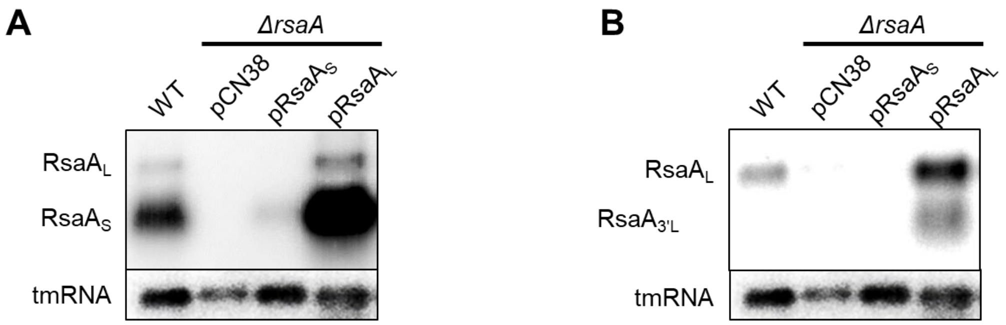

Figure S8. Accumulation of $RsaA_S$ in presence of $RsaA_L$.
(A) Northern blot of WT and *ΔrsaA* complemented with empty vector (pCN38), RsaA short form ($pRsaA_S$) or RsaA long form ($pRsaA_L$), sampled at an OD_{600} of 6 and probed for RsaA using a 5'-end probe to detect both short and long forms, with tmRNA probed as a loading control (n = 4).
(B) Northern blot using the same membrane as for Fig. S5A, probed for $RsaA_L$ and $RsaA_{3'L}$ only using a $RsaA_L$ 3'-end probe, and tmRNA (loading control) (n = 4).

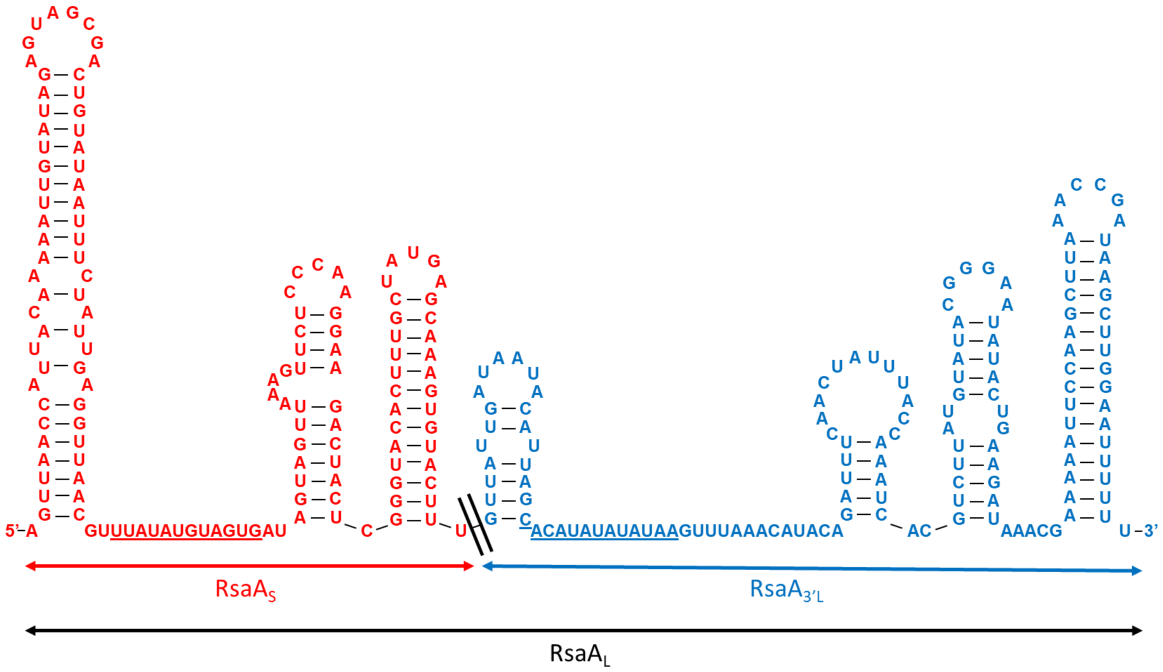

Figure S9. Simplified diagram of RsaA secondary structure with proposed cleavage site.
RsaA$_S$ (positions 1-144) is indicated in red, and RsaA$_{3'L}$ (positions 145-282) is indicated in blue. Nucleotide positions 1-144 were cloned into pCN38-rsaA$_S$ to express the short-form region of RsaA. Nucleotide positions 145-282 were cloned into pCN38-rsaA$_{3'L}$ to express only the long region of RsaA$_L$. The proposed cleavage site is indicated by the two parallel black lines. The predicted RsaA$_S$-RsaA$_{3'L}$ interaction regions, as described in Figure S9, are underlined.

```
                             15              27
                              |               |
                      5'-UGA..AUUAG   A         GUUUA..UUU-3'
RsaA_L-region sRNA                 CAC UAUAUAUAA
                                   ||| |||||||||
RsaA_S sRNA                        GUG AUGUAUAUU
                      3'-UAU..UGAUA             UGCAA..UGA-5'
                              |               |
                             73              62
```

Energy: -8.14 kcal/mol
Hybridisation Energy: -9.2 kcal/mol

Figure S10. Predicted RNA-RNA interaction between RsaA$_S$ and RsaA$_{3'L}$.
Predicted interaction between RsaA$_S$ (144bp) and RsaA$_{3'L}$ (138bp), as proposed by IntaRNA RNA-RNA interaction prediction software (9). The energy score (bold) of a predicted RNA-RNA interaction is the sum of the hybridisation free energy of the interacting sub-sequences, and the free energies required to unfold the interaction sites in both RNA molecules.

References

1. Herbert S, Ziebandt AK, Ohlsen K, Schäfer T, Hecker M, Albrecht D, Novick R, Götz F. 2010. Repair of global regulators in *Staphylococcus aureus* 8325 and comparative analysis with other clinical isolates. Infect Immun 78:2877–2889.
2. Le Lam TN, Morvan C, Liu W, Bohn C, Jaszczyszyn Y, Bouloc P. 2017. Finding sRNA-associated phenotypes by competition assays: An example with *Staphylococcus aureus*. Methods 117:21–27.
3. Coronel-Tellez RH, Pospiech M, Barrault M, Liu W, Bordeau V, Vasnier C, Felden B, Sargueil B, Bouloc P. 2022. sRNA-controlled iron sparing response in Staphylococci. Nucleic Acids Res 50:8529–8546.
4. Crosby HA, Schlievert PM, Merriman JA, King JM, Salgado-Pabón W, Horswill AR. 2016. The *Staphylococcus aureus* Global Regulator MgrA Modulates Clumping and Virulence by Controlling Surface Protein Expression. PLoS Pathog 12:e1005604.
5. Charpentier E, Anton AI, Barry P, Alfonso B, Fang Y, Novick RP. 2004. Novel cassette-based shuttle vector system for Gram-positive bacteria. Appl Environ Microbiol 70:6076–85.
6. Wiegand I, Hilpert K, Hancock REW. 2008. Agar and broth dilution methods to determine the minimal inhibitory concentration (MIC) of antimicrobial substances. Nat Protoc 3:163–175.
7. Romilly C, Lays C, Tomasini A, Caldelari I, Benito Y, Hammann P, Geissmann T, Boisset S, Romby P, Vandenesch F. 2014. A Non-Coding RNA Promotes Bacterial Persistence and Decreases Virulence by Regulating a Regulator in *Staphylococcus aureus*. PLoS Pathog 10:e1003979.
8. Wheeler DL, Barrett T, Benson DA, Bryant SH, Canese K, Chetvernin V, Church DM, Dicuccio M, Edgar R, Federhen S, Feolo M, Geer LY, Helmberg W, Kapustin Y, Khovayko O, Landsman D, Lipman DJ, Madden TL, Maglott DR, Miller V, Ostell J, Pruitt KD, Schuler GD, Shumway M, Sequeira E, Sherry ST, Sirotkin K, Souvorov A, Starchenko G, Tatusov RL, Tatusova TA, Wagner L, Yaschenko E. 2008. Database resources of the National Center for Biotechnology Information. Nucleic Acids Res 36:D13–D21.
9. Mann M, Wright PR, Backofen R. 2017. IntaRNA 2.0: enhanced and customizable prediction of RNA–RNA interactions. Nucleic Acids Res 45:W435–W439.
